# Retraction Note: Flowering season of vernal herbs is shortened at elevated temperatures with reduced precipitation in early spring

**DOI:** 10.1038/s41598-025-98207-5

**Published:** 2025-04-23

**Authors:** Bo Eun Nam, Jae Geun Kim

**Affiliations:** 1 Department of Biology Education, Seoul National University, Seoul, 08826 Republic of Korea; 2 Center for Education Research, Seoul National University, Seoul, 08826 Republic of Korea

Retraction of: *Scientific Reports* 10.1038/s41598-020-74566-z, published online 15 October 2020

The Editors have retracted this Article.

After publication of this Article, concerns were raised regarding the design of the study and the robustness of its central conclusions. Post-publication peer review has confirmed critical flaws, including the following:

- Available evidence does not confirm that elevated temperature and water stress conditions have been established;
- Purchased plant species have not been authenticated;
- The study has low statistical power given the small number of flowering plants per species per condition;
- Data have only been collected over a single flowering season.

The Editors therefore no longer have confidence in the conclusions presented.

Bo Eun Nam and Jae Geun Kim agree to this retraction.

